# Isolation and characterization of chromosomal markers in *Poa pratensis*

**DOI:** 10.1186/s13039-017-0307-7

**Published:** 2017-03-09

**Authors:** Yanyan Zhao, Feng Yu, Ruijuan Liu, Quanwen Dou

**Affiliations:** Key Laboratory of Adaptation and Evolution of Plateau Biota, Northwest Institute of Plateau Biology, the Chinese Academy of Sciences, Xining, 810008 China

**Keywords:** *Poa pratensis*, Repetitive sequence, Chromosomal markers

## Abstract

**Background:**

*Poa pratensis* L. is a turf grass and forage crop used worldwide. Being a facultative apomictic species, *P. pratensis* has a highly variable chromosome number. Chromosomal markers constitute a powerful tool for chromosome identification and for various aspects of genomic research. However, currently, no chromosomal markers are available for *P. pratensis*.

**Results:**

Four novel chromosome markers were isolated from a screen of Cot-1 DNA libraries, combined with fluorescence in situ hybridization (FISH) in *Poa pratensis*. Three tandemly repetitive sequences (*Pp*TR-1, *Pp*TR-2, and *Pp*TR-3) were characterized as subtelomeric. Monomers of 318 bp, 189 bp and 189 bp were identified in *Pp*TR-1, *Pp*TR-2, and *Pp*TR-3, respectively. One tandemly repetitive sequence (*Pp*CR-1) was shown to be centromeric or pericentromeric, and it had a monomer of 27 bp. The distribution patterns of *Pp*TR-1, *Pp*TR-2, and *Pp*TR-3 were highly conserved across different *P. pratensis* cultivars and in the distantly related *Poa* species, whereas *Pp*CR-1 was conserved across different *P. pratensis* cultivars, but less conserved across *Poa* species.

**Conclusion:**

In this study, we report the identification and characterization of four novel chromosomal markers in *P. pratensis*. These chromosomal markers are powerful tools for accurate assessment of chromosome count, genomic and phylogenetic analyses, as well as studies of apomixis in *P. pratensis.*

**Electronic supplementary material:**

The online version of this article (doi:10.1186/s13039-017-0307-7) contains supplementary material, which is available to authorized users.

## Background

*Poa pratensis* L. (Kentucky bluegrass), which belongs to the *Gramineae* family, is a perennial herbaceous plant with strong regenerative ability, high fecundity, cold resistance, drought tolerance, and rapid colonization [1]. It is used worldwide as a temperate turf grass and forage crop [2, 3]. Through facultative apomixis, this species can propagate diverse and odd ploidy levels, resulting in a wide range of chromosome numbers [4–6].

Traditionally, the ploidy levels in *Poa* were determined by conducting chromosome counts of root tip cells. However, given their relatively small size, high preponderance and morphological similarity, the chromosome number of *P. pratensis* is difficult to accurately quantify [7]*.* Flow cytometry is the commonly used method for the measurement of DNA content in somatic cells of *P. pratensis* to determine the ploidy level [8]. This approach has been used effectively in *P. pratensis* as an accurate method to evaluate ploidy level in a large number of individual plants [9, 10]. However, quantification of chromosome counts would still be indispensable, particularly, if confirmation of ploidy level is required [11].

The repetitive sequences are commonly used as probes in FISH (fluorescence in situ hybridization) assay for various applications, including identification of individual chromosomes, study of karyotype evolution, and screening for chromosome aberrations [12–14]. Among the 10 tested repetitive sequences, pTa535 was shown to be the most valuable, allowing A-genome chromosome identification and species discrimination across different diploid and polyploid wheat [12]. *Brachypodium pinnatum* chromosomes could be accurately identified using rDNA-based and species-crossing BAC-based FISH probes [13]. However, to date, no information on chromosomal markers is available for *P. pratensis.*

Cot-1 DNA is enriched with highly and moderately repetitive sequences. The labelled Cot-1 DNA could be used (as a probe) to localize heterochromatin in chromosomes [14]. A number of previous studies have shown that chromosomal markers could be developed through construction and screening of Cot-1 libraries [15–18]. For example, 11 tandemly repetitive sequences were identified from a Cot-1 library by FISH, followed by sequence analysis of alfalfa (*Medicago sativa*), and this approach was shown to be the most accurate to identify alfalfa chromosomes [18].

In this study, we first constructed a *P. pratensis* Cot-1 library and subsequently conducted FISH screening with labelled clones on mitotic chromosomes. The clones showing strong hybridization signals were further investigated and several distinct chromosomal markers were identified. In this paper, we discuss the application of these chromosomal markers for the determination of ploidy level, assessment of genome composition, phylogenetic analysis, and identification of different types of apomixis.

## Methods

### Plant materials

Three local *Poa* cultivars and five introduced *P. pratensis* cultivars were used in this study (Table 1). Seeds were germinated at room temperature. The germinated plants were transplanted to the pots and grown at 20 °C in an artificial climate chamber with 12 h light and 12 h darkness.

**Table 1:** Materials used in this study

No.	Cultivar	Source
1	*P. pratensis* ‘Qinghai’	Qinghai, China
2	Park	Jacklin, USA
3	Geronimo	DLF-pickseeds, USA
4	Rhythm	DLF-pickseeds, USA
5	Midnight	DLF-pickseeds,USA
6	Kentucky	Jacklin, USA
7	*P. pratensis* var. *anceps* ‘Qinghai’	Qinghai, China
8	*P. crymophila* ‘Qinghai’	Qinghai, China

### Cot-1 DNA library construction

The procedure for Cot-1 DNA library construction was adopted from Yu et al. [18], with minor modifications. Genomic DNA of *P. pratensis* ‘Qinghai’ was autoclaved at 120 °C for 8 min and digested with S1 nuclease for 18 min 50 S. Thereafter, the fragmented DNA was enriched for 100-bp to 400-bp fragments. Subsequently, purified Cot-1 DNA fragments were ligated into the pGEM-T easy vector. Transformed *E. coli* cells (DH5α) were identified by blue/white screening, as per the manufacturer’s instructions.

### Probe preparation

The 5S rDNA was amplified by polymerase chain reaction (PCR) using genomic DNA of *P. pratensis*, as described by Fukui et al. [19]. Inserts of the candidate clones from the constructed Cot-1 library were amplified by PCR, using T7 and SP6 primers. Purified PCR products of 5S rDNA and clone inserts were labelled with tetramethyl-rhodamine-5-dUTP (red) or fluorescein-12-dUTP (green) (Roche Diagnostics) by a random primer labelling method, described by Dou et al. [20]. The pWrrn (clone which contains fragments of wheat 45S rDNA) provided by Professor Tsujimoto (Tottori University, Japan) were labelled by a nick-translation method.

### Chromosome preparation

Root tips, with a length of approximately 1–2 cm, were excised and pre-treated in ice-cold water at 0 °C for 24 h, and fixed in ethanol:glacial acetic acid (3:1, v/v) for 24 h at room temperature. Each root tip was squashed in a drop of 45% acetic acid. The slides were kept at -80 °C in the Ultra-low temperature freezer for more than half an hour.

### FISH and microphotometry

FISH experiments were carried out as described by Dou et al. [21], with minor modifications. Samples on prepared slides were denatured in 0.2 M NaOH in 70% ethanol at room temperature for 10 min, rinsed in 100% cold ethanol (stored at minus 20 °C) for approximately 30 min, and allowed to air dry. The hybridization was carried out at 37 °C overnight in 10 uL of a mixture containing 10–15 ng of each labeled DNA probe, 5–10 mg of sonicated salmon sperm DNA, 50% formamide, 2 × SSC, and 10% dextran sulfate. After hybridization, the cover glass was removed gently, and the slide was washed with distilled water at room temperature. Chromosomes were stained with 4′,6-diamidino-2-phenylindole (DAPI). Slides were observed using a fluorescence microscope (DM R HC, Leica). Images were captured using a cooled CCD camera (Photometrics CoolSNAP) by means of Meta Imaging System (Universal Imaging Corporation). Finally, the images were optimized by contrast adjustment using Adobe Photoshop 6.0.

### DNA sequencing and data analysis

Cot-1 DNA cloned products were sequenced by Sangon Biotech Co., Ltd. (Shanghai, China). The DNAman software package (Lynnon Biosoft, Quebec, Canada) was used to analyse the sequence data. Sequence similarities were queried against the NCBI nucleotide database using BLASTN.

## Results

### Screening and characterization of Cot-1 clones using FISH

Cot-1 DNA is enriched for highly and moderately repetitive DNA sequences. The Cot-1 DNA clones contained highly repeated DNA sequences that often produced a discernible “block” or “dot” signal on chromosomes when visualized by FISH. Firstly, a total of 396 positive Cot-1 clones were screened from 477 clones. The PCR product sizes of the positive clones ranged from 500 to 300 bp. Secondly, 44 Cot-1 positive clones, which were randomly picked, were amplified by PCR and fluorescently labelled. Subsequently, the fluorescent probes made from the insert fragments were hybridized to *P. pratensis* interphase nuclei and mitotic metaphase chromosomes. The results showed that 14 clones, accounting for 31.8% of those tested, produced distinct “dot” or “block” hybridization signals on the chromosomes. To characterize the signals, we categorized the positive clones into two types, according to their chromosomal distribution (Table 2).

**Table 2:** Chromosomal distribution of clones from *P. pratensis* Cot-1 DNA library

FISH pattern	Clone number	Percentage of the total
Type1 (centromeric sites)	6	7.1%
Type2 (subtelomeric sites)	1,23,37,88,90,91,94,153,155,208,232,236,265	92.3%

Type 1 showed hybridization signals around the centromere and included 1 clone (7.1%). Type 2 showed hybridization signals on the subtelomeric or telomeric regions; most of the clones (92.3%) belonged to this group.

### Sequencing and characterization of the screened clones

All 14 clones that we screened were subjected to sequencing. To characterize the sequences of the inserts, we performed homology searches against the existing nucleotide sequences in the NCBI database. The search results revealed no significant sequence similarities. Sequence comparisons revealed that some screened clones had high homology to each other. Finally, data analysis revealed four unique sequences among the 14 positive clones, namely, clone 1, clone 6, clone 23 and clone 94.

### Obtaining whole repetitive monomers

**Fig. 1:**
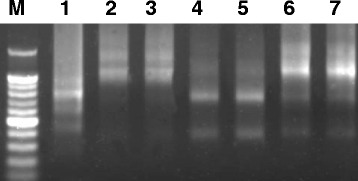
Patterns of PCR products amplified from designed primers (Table 3). 1, clone 94; 2 and 3, clone 23; 4 and 5, clone 6; 5 and 7, clone 1

The Cot-1 clones contained partial or complete sequences from the same repetitive sequence family. Thus, it was necessary to perform further experiments to determine the length of the repeated monomers. Additional PCRs were conducted using genomic DNA as the template. The oligonucleotide primer sequences were designed from the sequencing information for clone 6, clone 1, clone 23, and clone 94, using the Primer Premier 5.0 software (*PREMIER* Biosoft international, Canada) (Table 3). Electrophoresis of the PCR products of each pair of primers showed smearing, along with several intense amplification products (Fig. 1). This result implies a distribution pattern of tandem repeats of each tested monomer in the *P. pratensis* genome. A PCR product library was constructed and clones containing large fragments were sequenced.

**Table 3:** PCR primers used in monomer amplification in *P.pratensis*

Monomers	Sequence (5-- > 3')	Annealing temperature (°C)
Clone 6-F (*Pp*CR-1)R	ACCGTGAACTCTGCGTCG	53
Clone 6-R (*Pp*CR-1)F	TGGACTACCGACGCAGAGTTCAC	
Clone 1-F (*Pp*TR-1)R	AAGTTCTCAAGGTTTTACCTTCACC	55
Clone 1-R (*Pp*TR-1)F	GGACTACCGACGCAGAGTTCA	
Clone 23-F (*Pp*TR-2)R	AATCACGTCTTGTGACCGAG	55
Clone 23-R (*Pp*TR-2)F	GGCTCGGTCACAAGACG	
Clone 94-F (*Pp*TR-3)R	AAATTGATGCTTGACTAGTTGGTGA	53
Clone 94-R (*Pp*TR-3)F	CAGCAAATACACTACTCCAGC

A 751-bp PCR fragment was obtained with the primers for clone 6. Sequence analysis revealed 27-bp monomers, which were repeated 25 times. (KY618839, NCBI GeneBank)(Additional file 1: Figure S1). Clone 6 hybridized near centromeric regions of *P. pratensis* chromosomes in previous tests. We named this 27 bp repetitive monomer *Pp*CR-1. A 412-bp fragment was obtained with the primers for clone 1. Sequence analysis showed that the amplified fragment included one integrated 318 bp monomer unit and part of the second repeated motif (KY618838, NCBI GeneBank)(Additional file 1: Figure S2). A 698-bp fragment and a 689-bp fragment were obtained with the primers for clone 23 and clone 94, respectively (KY618840 and KY618841 , NCBI GeneBank)(Additional file 1: Figure S3 and S4). Three complete 189-bp monomers and two partial monomers were found in both sequences. Given that clones 1 23 and 94 were characterized as subtelomeric or telomeric in *P. pratensis* chromosomes by FISH, we designated the 318, 189, and 189 bp monomers derived from the aforementioned clones, as *Pp*TR-1, *Pp*TR-2, and *Pp*TR-3, respectively.

### Characterization of newly identified tandem repeats on mitotic chromosomes across *P. pratensis* cultivars

**Fig. 2:**
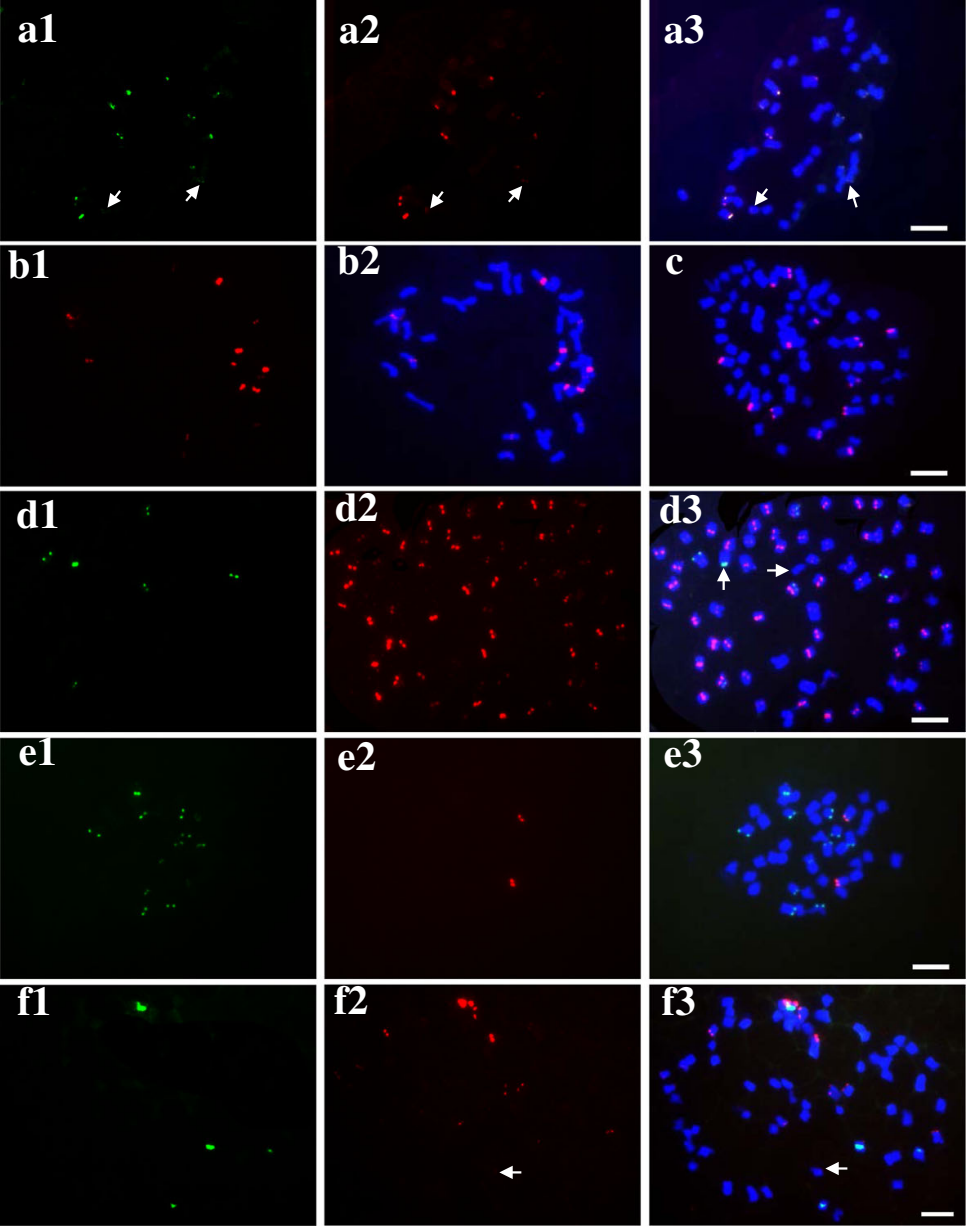
FISH patterns of mitotic chromosomes of *Poa pratensis* cultivars probed for repetitive sequences: **a1-a3** *PpTR*-1 (*green*), *Pp*TR-2 (*red*) in ‘Park’; **b1-b2** *Pp*CR-1 (*red*) in ‘Qinghai’; **c** *PpTR*-3 (*red*) in ‘Midnight’; **d1-d3** *Pp*TR-1 (*green*) and *Pp*CR-1 (*red*) in ‘Warrior’; **e1-e3** *PpTR*-1 (*green*) and *PpTR*-3 (*red*) in ‘Qinghai’; **f1-f3** *Pp*TR-1 (*red*) and 45S rDNA (*green*) in ‘Rhythm’. *Arrows* indicate the weak hybridization signals in all, except d3. *Arrows* in d3 indicate chromosomes without *Pp*CR-1 sites. Scale bar =10 μm

FISH analyses revealed that *PpCR*-1 was physically mapped near centromeric regions, whereas *Pp*TR-1, *Pp*TR-2 and *Pp*TR-3 were mapped to subtelomeric regions on mitotic chromosomes in the *P. pratensis* ‘Qinghai’ cultivar in a previous test (Fig. 2b and e). Due to the apomixis and diversity of *P. pratensis*, six *P. pratensis* cultivars, the Chinese cultivar *P. pratensis* ‘Qinghai’, and the American cultivars ‘Park’, ‘Geronimo’, ‘Rhythm’, ‘Midnight’, and ‘Kentucky’ were examined to assess the extent of evolutionary conservation and variability of the identified repeats between different individuals and across different cultivars. In addition to the four identified repeats, two repetitive DNAs, namely, 5S rDNA and 45S rDNA, which are found universally in plant genomes, were also used in this study as references. Single probes or probe cocktails of different combinations were used for FISH detection on mitotic chromosomes. The distribution information of each repeat was obtained from at least six different individuals of the each tested cultivar.

The repeats, *Pp*TR-1 and *Pp*TR-2, were co-localized in chromosomes of the cultivar ‘Qinghai’. Further tests also showed the co-localization of *Pp*TR-1 and *Pp*TR-2 in other *P. pratensis* cultivars (Fig. 2 a). The chromosomal distribution shared between *Pp*TR-1 and *Pp*TR-2 is also shared between different individuals or different cultivars in *P. pratensis*.

**Fig. 3:**
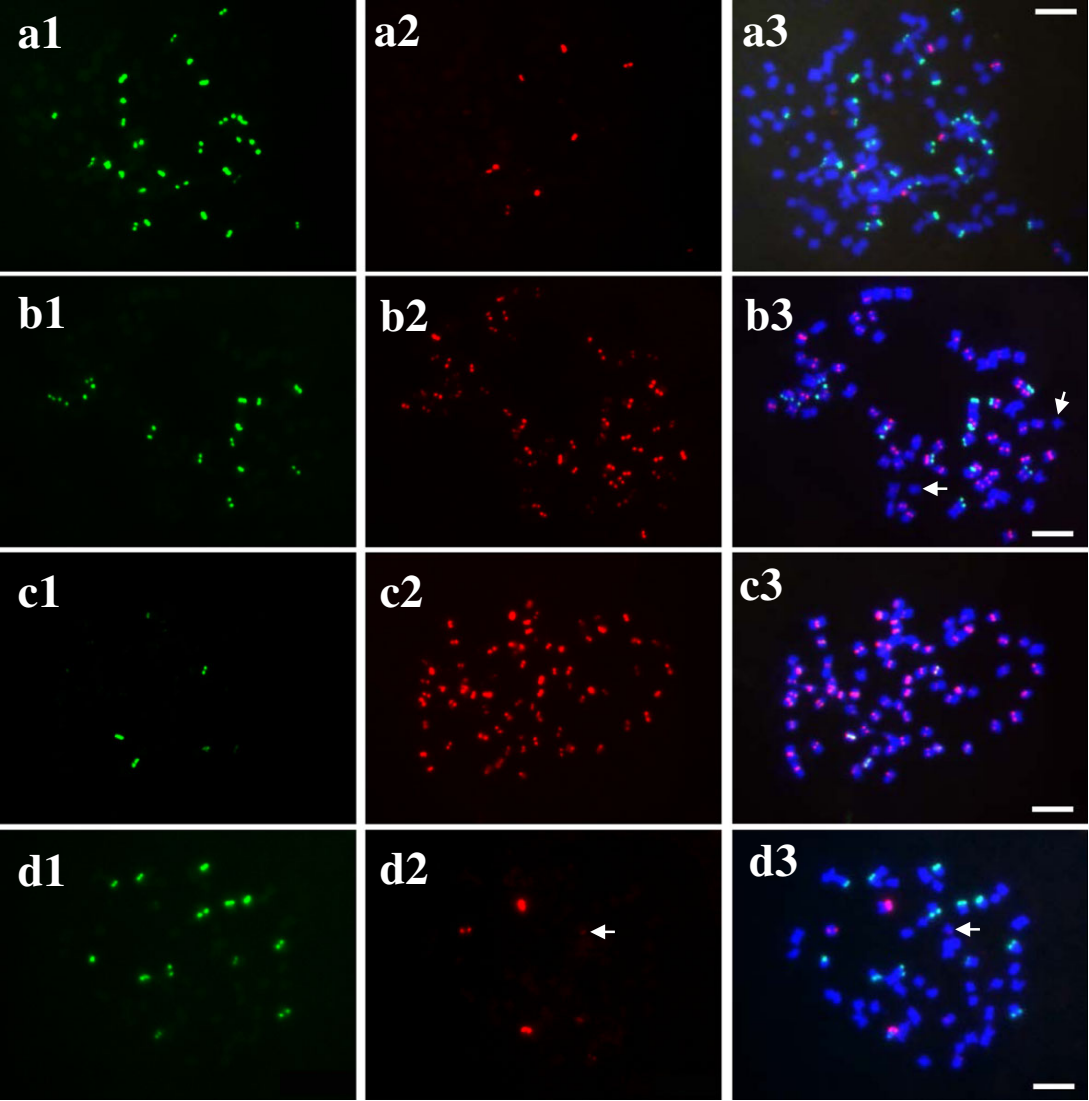
FISH patterns of mitotic chromosomes of *Poa pratensis* cultivars probed for repetitive sequences: **a1-a3** *PpTR*-1 (*green*) and 5S rDNA (*red*) in ‘Qinghai’; **b1-b3** *Pp*CR-1 (*red*) and *Pp*TR-3 (*green*) in ‘Warrior’; **c1-c3** *Pp*CR-1 (*red*) and 45S rDNA (*green*) in ‘Park’; **d1-d3** *Pp*TR-3 (*green*) and 5S rDNA (*red*) in ‘Kentucky’. *Arrows* in b3 indicates chromosomes without *Pp*CR-1 sites. *Arrows* in d2 and d3 indicate the weak hybridization signals. Scale bar =10 μm

The FISH patterns showed that *Pp*CR-1 hybridized on the pericentromeric regions of nearly all chromosomes (chromosomes with hybridization signals corresponding to *Pp*CR-1) across different cultivars, and each targeted chromosome carried only one *Pp*CR-1 hybridization site (Fig. 2 d; Fig. 3b and c). Occasionally, a very small number of chromosomes presenting hybridization signals on the ends of the chromosomes were observed in a few individuals of some cultivars. Because of the highly variable karyotypes of *P. pratensis*, the few chromosomes with a subtelomeric-like distribution of *Pp*CR-1 were mostly telosomic, originating from centromeric fission. Thus, pericentromeric distribution of *Pp*CR-1 in *P. pratensis* was confirmed*.* The *Pp*TR-1 was physically mapped onto subtelomeric regions of all chromosomes (chromosomes with hybridization signals corresponding to *Pp*TR-1) across different cultivars (Fig. 2d, e, f; Fig. 3a). In most cases, each targeted hybridized chromosome carried a single *Pp*TR-1 site, but one to two chromosomes carrying two *Pp*TR-1 signals were also observed in a few individuals in ‘Qinghai’ and ‘Park’. The single hybridization of *Pp*TR-3 was exclusively detected on the subtelomeric regions of some chromosomes across all cultivars (Fig. 2c, e; Fig. 3b, d). Most 45S rDNA sites were located on the pericentromeric regions, and 5S rDNA sites were detected in various regions, namely, the pericentromeric, intercalary, or subtelomeric sections across cultivars (Fig. 3c, d).

Highly variable chromosome numbers, ranging from 35 to 112, were observed among individuals and cultivars. The hybridization sites varied from 10 to 89, 7 to 23, and 1 to 23 for *Pp*CR-1, *Pp*TR-1, and *Pp*TR-3, respectively, whereas variable sites from 3 to 11 and 3 to 9 for 45S rDNA and 5S rDNA, respectively, were detected across 6 cultivars (Table 4).

**Table 4:** Cytogenetic characteristics of *P. pratensis* cultivars and related species

	*P. pratensis*						Relate species	
	*P. pratensis* ‘Qinghai’	Park	Kentucky	Rhythm	Midnight	Geronimo	*P. pratensis* var. anceps ‘Qinghai’	*P. crymophila* ‘Qinghai’
Chr. No. average	45	60	75	66	70	60	55	28
Chr. No. range	35–112	49–86	52–88	49–98	42–102	32–71	49–63	28
45S sites range (Chr. No. range)	4–8 (35–49)	3–8 (49–86)	5–8 (65–88)	4–9 (56–91)	5–11 (42–102)	3–7 (32–70)	7–9 (49–63)	6–9
5S sites range (Chr. No. range)	5–9 (35–112)	4–8 (49–86)	3–8 (65–84)	4–5 (49–77)	3–8 (42–102)	3–5 (32–70)	6–9 (49–63)	4–5
*Pp*CR-1 sites range (Chr. No. range)	10–19 (32–45)	47–78 (49–79)	69–86 (70–88)	59–89 (63–91)	54–80 (56–84)	45–68 (46–71)	19–24 (49–63)	—
*Pp*TR-1 sites range (Chr. No. range)	12–18 (35–42)	14–23 (49–84)	12–17 (63–85)	11–18 (56–98)	7–16 (56–80)	11–14 (46–71)	5–9 (49–56)	12–18
*Pp*TR-3 range range (Chr. No. range)	1–3 (42–112)	8–20 (49–78)	14–18 (52–81)	12–16 (49–82)	9–17 (50–84)	8–23 (38–70)	2–4 (49–63)	1–2

Chr. Indicates chromosome and No. indicates number

The connection between the number of hybridization sites of each repeat and the variation in total chromosome number was not distinctly revealed due to the complicated facultative apomixis of species of *P. pratensis*. In this study, the percentage of the number of hybridization sites to total chromosome number and the coefficient of variation (CV) were parameters used to more accurately evaluate the variability and conservation of repeat distribution across cultivars. The statistical analyses showed that the cytogenetic characters of ‘Qinghai’ were significantly different from those of the other cultivars (Table 5). The ‘Qinghai’ cultivar showed the highest percentage of 5S rDNA and *Pp*TR-1 and lowest percentage of *Pp*TR-3, compared with the other cultivars. In addition, the CV of 5S rDNA, *Pp*TR-1, and *Pp*TR-3 in ‘Qinghai’ were higher than those of the others. These observations are likely due to ‘Qinghai’ being genetically distant from the other cultivars, and that the samples were collected from wild populations, where nearly no breeding selection was imposed. The percentages of Pp*TR*-1 and Pp*TR*-3 varied from 17.2 to 27.4%, and 18.8 to 26.0%, respectively, while the CV of *PpTR*-1 and Pp*TR*-3 varied from 7.7 to 18.1% and from 8.6 to 20.0%, respectively, in cultivars from the USA. These results suggest that approximately 20% of all chromosomes in individuals from American cultivars can be estimated to carry hybridization sites for *Pp*TR-1 or *Pp*TR-3. Furthermore, the distribution of *Pp*CR-1 showed the highest percentage, 97.0 to 98.2%, and the lowest CV, 1.3 to 1.7%, across cultivars from the USA. These results imply that nearly 100% of chromosomes carry one *Pp*CR-1 hybridization signal and that the number of *Pp*CR-1 signals can often represent the total chromosome number in individuals of American cultivars.

**Table 5:** Statistics results of the percentage of hybridization sites and those of CV across cultivars

	*P. pratensis* ‘Qinghai’	Park	Kentucky	Rhythm	Midnight	Geronimo	*P. pratensis* var. anceps ‘Qinghai’	*P. crymophila* ‘Qinghai’
45S (%) CV(%)	8.67 19.07	9.02 19.05	8.42 11.35	7.44 17.71	12.06 13.92	9.28 9.38	13.8 10.71	26.79 13.98
5S (%) CV(%)	13.69 28.42	8.29 17.30	8.04 27.54	7.71 19.64	8.12 16.85	7.71 11.29	11.93 11.61	14.88 9.80
*Pp*CR-1(%) CV(%)	40.45 15.27	98.19 1.37	97.08 1.57	96.51 1.71	97.5 1.72	96.96 1.73	41.47 9.71	—
*Pp*TR-1 (%) CV(%)	37.30 16.48	27.41 7.65	19.48 16.15	18.49 8.05	17.17 18.12	20.00 13.25	13.88 20.73	54.17 13.45
*Pp*TR-3 CV(%)	3.45 60.52	18.76 19.00	22.43 15.40	23.11 8.55	20.13 12.43	25.93 19.93	6.14 22.6	4.76 38.74

### Characterization of the repeats in *P. pratensis* related species

**Fig. 4:**
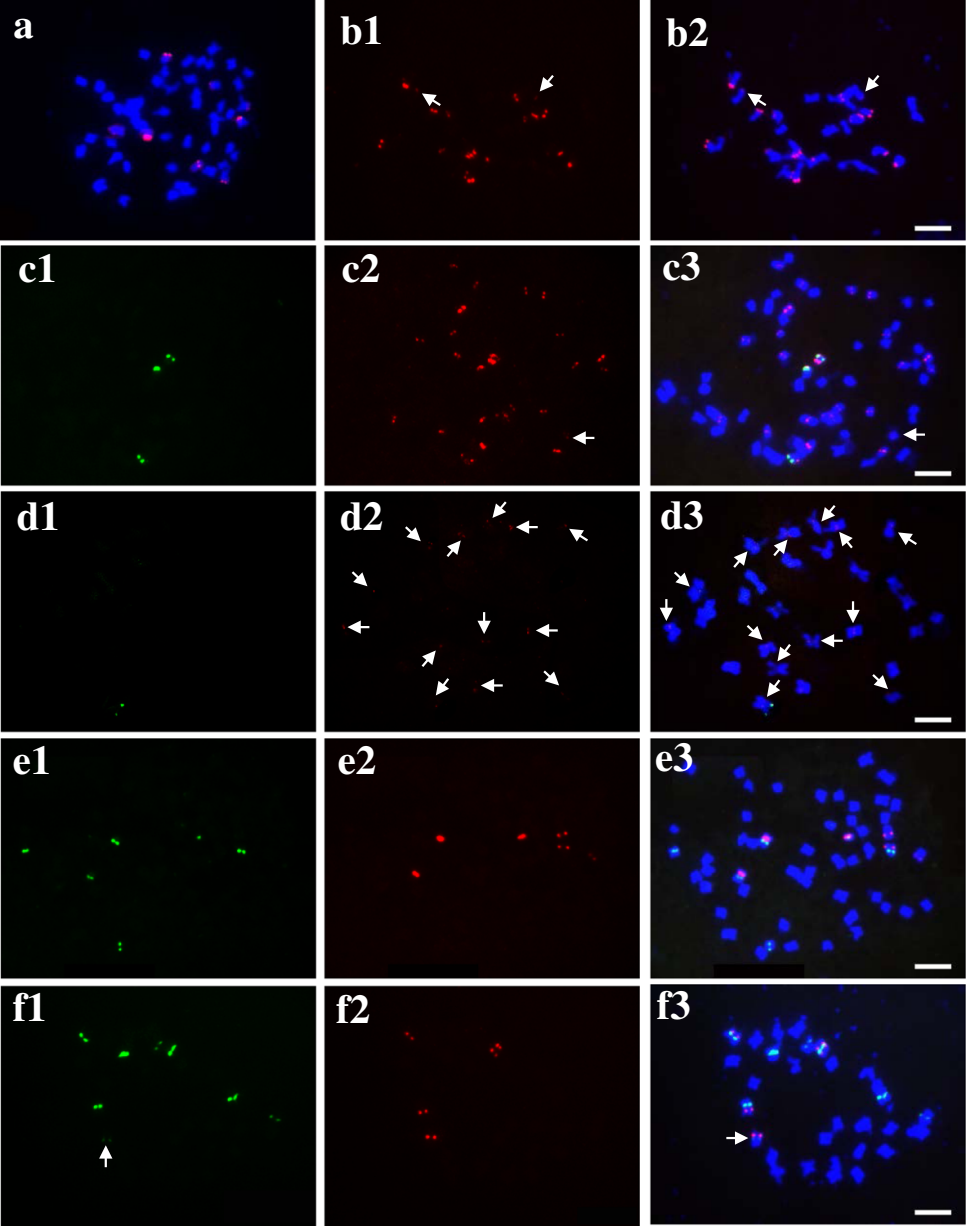
FISH patterns of mitotic chromosomes of *Poa pratensis* var. anceps ‘Qinghai’ (a,c and e) and *P. crymophila* ‘Qinghai’ (b, d, and f) probed with: **a** and **b** *Pp*TR-1 (red); **c** and **d** *Pp*CR-1 (red) + *Pp*TR-3 (green); **e** and **f** 45S rDNA (green) +5S rDNA (red). Arrows indicate the weak hybridization signals. Scale bar =10 μm

*P. pratensis* var. anceps ‘Qinghai’ presented more similar FISH patterns to *P. pratensis* ‘Qinghai’ for the tested repeats than to other cultivars (Fig. 4 a, c, e). However, variations between *P. pratensis* ‘Qinghai’ and *P. pratensis* var. *anceps* ‘Qinghai’ were still observed. *P. pratensis* ‘Qinghai’ showed a higher percentage of *Pp*TR-1 and a lower percentage of *Pp*TR-3 than *P. pratensis* var. anceps ‘Qinghai’ (Table 5).

The cytological characteristics of *P. crymophila* ‘Qinghai’ were distinctly different from those of *P. pratensis*. The stable chromosome number of 28 identified across the individuals implies that no apomixis occurs in this species (Table 5). Although faint *Pp*CR-1 hybridization signals were occasionally detected in a few chromosomes, clear hybridization sites of *Pp*TR-1 and *Pp*TR-2 were physically mapped exclusively on sub-telomeric regions on a few chromosomes (Fig. 4b, d, f).

## Discussion

### Variability and conservation of four newly identified chromosomal markers

The repeats *Pp*TR-1, *Pp*TR-2, and *Pp*TR-3 were clearly detected on subtelomeric chromosome regions across different *P. pratensis* cultivars or even other related *Poa* species, although large variations of the number of hybridization signals were identified among cultivars or species. Co-localized distribution was revealed between *Pp*TR-1 and *Pp*TR-2. Co-localization of a few repetitive DNAs was frequently identified in different genomes of the genus *Hordeum* [22, 23]. Although *Pp*TR-3, like *Pp*TR-1 and *Pp*TR-2, was physically mapped in the subtelomeric regions, the discrepancy in the sites between *Pp*TR-3 and *Pp*TR-1 or *Pp*TR-2 implies that their distribution was close rather than intermingled. Furthermore, the exclusive distribution of *Pp*TR-1, *Pp*TR-2 and *Pp*TR-3 not only in the closely related species *P. pratensis* var. *ancep* but also the distantly related species *P. crymophila* suggests that these sub-telomeric repeats may be highly conserved across different species in the genus *Poa*. A wide distribution of a 120-bp repeat across many different diploid and polyploid species was detected within the Triticeae tribe, and sequence similarity analysis of the repeat suggested that no characteristic genome- or species-specific variants developed during the evolution of the extant genomes [24]. This fact implies that the repeats *Pp*TR-1, *Pp*TR-2 and *Pp*TR-3, being present in the genome of common ancestral species of *Poa*, may have evolved in a similar way as the 120-bp repeat. *Pp*CR-1 is a mini-satellite DNA and was physically mapped at the pericentromeric regions. Plant centromere sequences are characterized by long arrays of highly repetitive satellite sequences that are interspersed frequently with centromeric retrotransposons [25, 26]. In this study, no other repetitive sequences around the centromeres were identified in *P. pratensis*. This result suggests that *Pp*CR-1 may be the main component of the centromeric repetitive sequence. Unlike *Pp*TR-1, *Pp*TR-2 or *Pp*TR-3, *Pp*CR-1 produced only faint and blurry hybridization in the pericentromeric regions of the distantly related species, *P. crymophila*. It can be inferred that *Pp*CR-1 may be more species-specific and may have evolved more rapidly than the subtelomeric repeats.

### Chromosome counting assisted by chromosomal markers

The chromosome number of *P. pratensis* is highly variable, with polyploidy and aneuploidy ranging from 2n = 28 to 154 [3–7]. The overlapped or agglomerated chromosomes in a mitotic chromosome-spread always make chromosome counting difficult, particularly for high-ploidy samples. Ideal chromosomal markers that are highly correlated with chromosome number would facilitate accurate chromosome counting. In this study, chromosomes with *Pp*TR-1, *Pp*TR-2, and *Pp*TR-3 were proportionally detected across different *P. pratensis*. Although total chromosome numbers could be estimated using the number of the chromosomal markers, the variability in relative percentages reduces accuracy. *Pp*CR-1 was detected in nearly all chromosomes (in more than 96% of all chromosomes) in all investigated cultivars except *P. pratensis* ‘Qinghai’, and *Pp*CR-1 exhibited the lowest percentage of variation. This result suggests that the total chromosome number can be accurately determined by counting *Pp*CR-1 hybridization signals. *Pp*CR-1 is a useful chromosomal marker for chromosome number determination in *P. pratensis*.

### Phylogenetic and genome research with chromosomal markers

A basic genome with a chromosome number of seven was determined in *P. pratensis*, despite a highly variable number of chromosomes [6]. Observation of the occurrence of apomixis and the presence of “group segregation” in *P. pratensis* in Missouri, USA suggested an allopolyploid origin of the species [27]. Analysis of chloroplast and nuclear gene sequence data supports the idea that *P. pratensis* is, at least, partly allopolyploid [28]. In this study, *P. pratensis* from Qinghai, China, showed distinctly fewer *Pp*CR-1 and *Pp*TR-3 sites, compared with cultivars from the USA. This result implies a possible large genomic divergence between these cultivars. The genome composition of Qinghai *P. pratensis* presents as more likely to be allopolyploid than those of cultivars from the USA. This observation means that the ability to produce polyploidy with facultative apomictic reproduction may make the genome composition of *P. pratensis* more diverse and complex. Chromosomal markers, using comparative FISH analyses, would be helpful in elucidating plant genome composition in phylogenetic analysis. Comparative karyotyping of *Brachypodium pinnatum* was done using cross- species BAC-FISH [13]. Phylogenetic relationship of A genome between diplopid and tetraploid wheat was exclusively uncovered by identification of all A-genome chromosomes, using a set of chromosomal markers [12]. Thus, comparative cytogenetic analysis of the many other *Poa* species using the chromosomal markers identified in this study may provide valuable information about the origin of *P. pratensis*.

### Evaluation of reproductive mode and analysis of inheritance of apomixis assisted by chromosomal markers

*Poa pratensis* has been described as an aposporous and pseudogamous facultative apomictic species [29]. Flow cytometry analysis has revealed that five routes of seed formation and four reproductive pathways define the reproductive mode of *P. pratensis* plants in *P. pratensis* cultivars and core accessions [10, 20]. The inheritance of apomixis in *P. pratensis* has been confirmed in a five-locus model with differences in gene expressivity and penetrance [30]. However, which individual gene is linked to apospory and/or parthenogenesis still remains elusive. Despite their small size and high count, the identification of these chromosomes could be greatly improved using newly identified chromosomal markers, combined with 5S rDNA and 45S rDNA. This fact suggests that the reproductive mode of *P. pratensis* plants can be molecularly and cytogenetically studied by comparing the cytotypes of the parents and progeny, and apomictic inheritance analysis may be aided by finding apomixis-associated chromosomes or chromosome regions.

## Conclusions

In this study, we report four novel chromosome markers that were developed by screening Cot-1 DNA libraries, combined with FISH assay in *Poa pratensis*. Three (*Pp*TR-1, *Pp*TR-2, and *Pp*TR-3) were characterized as subtelomeric, and one (*Pp*CR-1) was centromeric or pericentromeric. The chromosomal markers, *Pp*TR-1, *Pp*TR-2, and *Pp*TR-3, were stably detected across different *P. pratensis* cultivars and in the distantly related *Poa* species. However, *Pp*CR-1 was conserved across various *P. pratensis* cultivars but less conserved across *Poa* species. These chromosomal markers will be powerful tools for chromosome counting, genomic and phylogenetic analyses, and studies of apomixis in *P. pratensis.*

## Additional file

Additional file 1: Figure S1.
*Pp*CR-1 monomer hunting from the amplified sequence by designated primers of clone 6. **Figure S2**. *Pp*TR-1 monomer hunting from the amplified sequence by designated primers of clone 1. **Figure S3**. *Pp*TR-2 monomer hunting from the amplified sequence by designated primers of clone 23. **Figure S4**. *Pp*TR-3 monomer hunting from the amplified sequence by designated primers of clone 94. (DOC 44 kb)

## Abbreviations

CR: Centromeric repeat
*Pp*: *Poa pratensis*
TR: Telomeric repeat
