# Supplementary material for: Isolation and characterization of chromosomal markers in Poa pratensis
Source: Mol Cytogenet. 2017 Mar 9;10:5. doi: 10.1186/s13039-017-0307-7 (PMC5345224; doi:10.1186/s13039-017-0307-7)
Supplement: Additional file 1: Figure S1. — PpCR-1 monomer hunting from the amplified sequence by designated primers of clone 6. Figure S2. PpTR-1 monomer hunting from the amplified sequence by designated primers of clone 1. Figure S3. PpTR-2 monomer hunting from the amplified sequence by designated primers of clone 23. Figure S4. PpTR-3 monomer hunting from the amplified sequence by designated primers of clone 94. (DOC 44 kb) [file 13039_2017_307_MOESM1_ESM.doc]

Seq1 **······························ ···**- - - - - - - - - - AG 29

Seq2 - - - - - - - - - - - - - - - - - -- - - - - - - - - - - -GG 29

Seq3 TACCGTGAACTCTGCGTCGGTAGTCCA 27

Seq4 - - - - - - - - - - - - - - - -- - - - - - - - - - - - - - - 27

Seq5 ACGG- - - - - - - - - - - - - - - - - - - - - - - - - - - - - - 31

Seq6 ACCTTC- - - - - - - - - - - - - - - - - - - - - - - - - - - - - - 32

Seq7 **·** - - - - - - - - - - - - - - - - - - - - - - - - - - - - - 26

Seq8 ACGGC - - - - - - - - - - - - - - - - - - - - - - - - - - - - - C 32

Seq9 - - - - - - - - - - - - - - - - - - - - - - - - - - - - - - 27

Seq10 - - - - - - - - - - - - - - - - - - - - - - - - - - - - - - 27

Seq11 ACGG - - - - - - - - - - - - - - - - - - - - - - - - - - - - - - 31

Seq12 AC - - - - - - - - - - - - - - - - - - - - - - - - - - - - - **·** 28

Seq13 **·** - - - - - - - - - - - - - - - - - - - - - - - - - - - - - 26

Seq14 ACCT - - - - - - - - - - - -- - - - - - - - - - - - - - - - - - 32

Seq15 - - - - - - - - - - - - - -- - - - - - - - - - - - - - - - 27

Seq16 - - - - - - - - - - - - - - - -- - - - - - - - - - - - - - 27

Seq17 - - - - - - - - - - - - - - - -- - - - - - - - - - - - - - 27

Seq18 - - - - - - - - - - - - - - - -- - - - - - - - - - - - - - 27

Seq19 - - - - - - - - - - - - - - - -- - - - - - - - - - - - - - 27

Seq20 C - - - - - - - - - - - - - - - -- - - - - - - - - - - - - - 28

Seq21 - - - - - - - - - - - - - - - -- - - - - - - - - - - - - - 27

Seq22 - - - - - - - - - - - - - - - -- - - - - - - - - - - - - - -AC 29

Seq23 - - - - - - - - - - - - - - - -- - - - - - - - - - - - - - 27

Seq24 - - - - - - - - - - - - - - - -- - - - - - - - - - - - - - 27

Seq25 ACGG - - - - - - - - - - - - - -- - - - - - - - - - - - - - - - AGTA 35

Seq26 GTCCAG - - - - - - - - - - - - - - - -- - - - - - - - - - - - - - 33

Seq27 - - - - - - - - - - - - - - - -- - - - - - - - - **·········** 24

**Figure S1 *Pp*CR-1 monomer hunting from the amplified sequence by designated primers of clone 6.**

Seq1 AAGTTCTCAAGGTTTTACCTTCACCGTGAACTCTGCGTCG 40

Seq2 AAGTTCTCAAGGTTTTACCTTCACCGTGAACTCTGCGTCG 40

Seq1 GTAGTCCACGGTGACGCGGCCGCCACGGTACCTAAACGGC 80

Seq2 GTAGTCC**······················································ ············** 80

Seq1 TCGGTCACAAGACTTGATTGGTACAGACATGATCTAAATA 120

Seq2 **································· ····················· ························** 120

Seq1 GCCCACCCTAACTTTCTATGTGCTACTCATGTTTTGATCG 160

Seq2 **································· ····················· ·····················** 160

Seq1 GCTAATCTGGAGGGTGGGTGGCCGCAGACATCTTCGAAGG 200

Seq2 **································· ·············································** 200

Seq1 AAGTGTAGGATTTCACCGAAACGAACGTTGCTCTCTAGCT 240

Seq2 **································ ·········· ··································** 240

Seq1 TACGATAAGCGAATTTTAGAAAAATCTCCATCGGGCCAAT 280

Seq2 **··········································· ·································** 280

Seq1 TTTGTAAATCTACTTTCTCTAGGGGTGTGGCATGTCATTC 320

Seq2 **··········································· ·································** 320

Seq1 TAGAGCCATGAAAAAAACACTAGCTCAATCGGGGTCCGGA 360

Seq2 **···································· ··········································** 360

Seq1 CGGCA 365

Seq2 **··········** 365

**Figure S2 *Pp*TR-1 monomer hunting from the amplified sequence by designated primers of clone 1.**

Seq1 **···············································································** 40

Seq2 CCGGTGTAGAAACTAGTCGATTGAGGCACCATTTACTCAG 40

Seq3 T- - - - - - - - - - - - - - - - - - -T- - - - - - - - - - T- - - - - - - - -T- - - - - 40

Seq4 C **·** - - - - - - - - - - - - - - - - - - - - - - - - - - - - - - - - - - - - - - - - - - - 39

Seq5 C - - - - - - - - - - A **·** - - - - - - - - - - - - - - - T- - - - - - - - - - -T - - - G 40

Seq1 **·············································································** 80

Seq2 TTTCTCTCAGGGCCTGGGGTTCAAATTGATGCTTGACTAG 80

Seq3 - - -- - - - - - - - - - - - - - - - - - - - - - - - - - - - - - - - - - - - - - - - - - - 80

Seq4 - - -- - - - - - - - - - - - - - - - - - - - - - - - - - - - - - - - - - - - - - - - - - - 80

Seq5 - - - - - - - - - - - -**··························································** 80

Seq1 **·····························································**- - - - - - - - - - - 120

Seq2 TTGGTGAACAAGCTGGAGTAGTGTATTTGCTGACCTGGAG 120

Seq3 - - - - - - - - - - - - - - - - - - - - - - - - - - - - - - - - - - - - -A- - - - - - - - 120

Seq4 - - - - - - - - -G- - - - - - - - - - - - - - - - - - - - - - - - - - -A- - - - - - - - 120

Seq5 **············································································ ·** 120

Seq1 - - -- - - - - - - - - - - - - - - - - - - - - - - - - - - - - - - - - - - - - - - - - - - 160

Seq2 GCATTTTGAATAAACAGGTTGTCGCGCGAAATGAAAACTG 160

Seq3 T- - - - - - - - - - - - - - - - - - - - - - - - - - - - -T- - - - - - - - - - - - - - -- 160

Seq4 - - -- - - - - - - - - - - - - - - - - - - - - - - - - - - - - - - - - - - - - - - - - - - 160

Seq5 **················································································** 160

Seq1 - - - - - C- - - - - - - - - - - - - - - - - - - - - - - - A- - 189

Seq2 AGTCCTAGAAACGGCCTCTTTAACGAGCG 189

Seq3 - - - - - - - - - - - - - - - - - - - - - - - - - - - - - - - - - -C 190

Seq4 - - - - - - - - - - - - - - - - AT- - - - - - - - - - - - - - - - 189

Seq5  **··························· ······················· ··· ··** 189

**Figure S3 *Pp*TR-2 monomer hunting from the amplified sequence by designated primers of clone 23.**

Seq1 **··············································································**  40

Seq2 CTCCTGGTCAGCAAATACACTACTCCAGCTTCTTCACCAA 40

Seq3 - - - - - - - - - - - - - - - - - - - - - - - - - - - - - - - - - - - - - - - - - - - - - - 40

Seq4 - - - --A - - - - - - - - - - - - - - - - - - - - - - - - - - - - - -G - - - - - - - - - 40

Seq5 CTCCTGGTC**················································· ············** 40

Seq1 **····················································** - - - - - - - - - - - - - - - 80

Seq2 CTAGTCAAGCATCAATTTGAACCCCAGGCCCTGAGAGAAA 80

Seq3 - - - - - - - - - - - - - - - - - - - - - - - - - - - - - - - - - - - - - - --- - - - - - - 80

Seq4 - - - - - - - - - - - - - - - - - - - - - - - - - - - - - - - - - - - - - - - - - - - - - - 80

Seq5 **·············································································· ·** 80

Seq1 - C- - - -A- - - - - - - - - - - -A - - - - - - - - - - - - - - -A - - - - - - - - - 120

Seq2 CTGAGTAAATGGTGCCTCAATCGACTAGTTTCTACACCGG 120

Seq3 - - - - - - - - - - - - - - - - - - A - - - - - - A- - - - - - - - - - - - - - - --CA 120

Seq4 - - - - - - - - - - - - - - - - - - - - - - - - - - - - - - - - - - - - - - -- - - - - - - 120

Seq5 **·················································· ···························· ·** 120

Seq1 - - - - - - - - - - - - - - - AT - - - - - - - - - - - - - - - - - - - - - - - - - - - T 160

Seq2 CGCTCGTTAAAGAGGCCGTTTCTAGGACTCAGTTTTCATA 160

Seq3 - - - - - - - - - - - - - - - - - - - - - -- - - - - - - - - - - - - - - - - - - - - - - T 160

Seq4 T - - - - - - - - - - - - - - - - - - - - - - - - - G - - - - - - - - - - - - - - - - -T 160

Seq5 **··············································································** 160

Seq1 - - - - - - - - - - - - - - C - - - - - - - - - - - - - - - - C 189

Seq2 TCGCGCGACAACATGTTTATTCAAAATGA· 189

Seq3 - - - - - - - - - - - - - - C - - - - - - - - - - - - - - - - C 189

Seq4 - - - - - - - - - - - - - - C - - - - - - - - - - - - - - - - C 189

Seq5 **··················································· ·····** 189

**Figure S4 *Pp*TR-3 monomer hunting from the amplified sequence by designated primers of clone 94.**
